# Association of estimated glomerular filtration rate and in-hospital mortality in patients hospitalized for heart failure: a retrospective cohort study

**DOI:** 10.3389/fcvm.2026.1780798

**Published:** 2026-06-09

**Authors:** Yueriyeti Aierken, Gulburak Taalaibek kyzy, Meng Wei, Yanmei Lu, Jianghua Zhang, Zukela Tuerhong, Baopeng Tang, Xianhui Zhou

**Affiliations:** 1 Department of Cardiac Pacing and Electrophysiology, the First Affiliated Hospital of Xinjiang Medical University, Urumqi, Xinjiang, China; 2 Xinjiang Key Laboratory of Cardiac Electrophysiology and Cardiac Remodeling, the First Affiliated Hospital of Xinjiang Medical University, Urumqi, Xinjiang, China; 3 School of Public Health, Xinjiang Medical University, Urumqi, Xinjiang, China

**Keywords:** estimated glomerular filtration rate, heart failure, in-hospital mortality, nonlinear relationship, retrospective cohort study

## Abstract

**Background:**

Assessment of mortality risk in hospitalized patients with heart failure (HF) constitutes a core tenet of clinical management. Renal function, as quantified by estimated glomerular filtration rate (eGFR), is a key prognostic factor for adverse outcomes; however, its independent association with in-hospital mortality has not been fully elucidated. We quantify the independent prognostic impact of eGFR on the risk of in-hospital mortality and to characterize its underlying nonlinear relationship.

**Methods:**

We retrospectively enrolled 14,591 patients hospitalized for HF at a tertiary care center (2009–2024). eGFR was calculated via the Chronic Kidney Disease Epidemiology Collaboration (CKD-EPI) equation and categorized into five categories: ≥90, 60–89, 45–59, 30–44, and <30 mL/min/1.73 m^2^. The primary end point was all-cause in-hospital mortality. Multivariable logistic regression with stepwise covariate adjustment, subgroup analyses, and restricted cubic spline (RCS) modeling were performed to evaluate the association between eGFR and mortality.

**Results:**

In-hospital mortality rates increased progressively across eGFR strata: 5.77%, 6.37%, 8.19%, 11.72%, and 10.93%, respectively. After full covariate adjustment (Model 3), compared with eGFR ≥ 90 mL/min/1.73 m^2^, eGFR 45–59 and 30–44 mL/min/1.73 m^2^ were independently associated with a higher in-hospital mortality risk (OR 1.27, 95% CI 1.02–1.58, *P* = 0.036; OR 1.36, 95% CI 1.07–1.75, *P* = 0.014). Subgroup analysis showed that patients with eGFR < 60 mL/min/1.73 m^2^ had a 37% higher risk of in-hospital mortality (OR1.37, 95% CI 1.19–1.58, *P* *<* 0.001). A significant interaction was observed between eGFR and New York Heart Association (NYHA) functional class (*P* for interaction <0.001), with the risk of in-hospital mortality being significantly amplified in patients with NYHA class Ⅲ (OR 1.75) and class Ⅳ (OR 2.13). RCS analysis revealed a nonlinear association between eGFR and in-hospital mortality risk (*P* for nonlinearity <0.001), with a critical inflection point at approximately 60 mL/min/1.73 m^2^; below this threshold, the risk of in-hospital mortality increased sharply.

**Conclusions:**

Reduced eGFR is a strong, independent prognostic factor for in-hospital mortality in patients hospitalized for HF, particularly in those with New NYHA class III/IV. The risk of in-hospital mortality rises steeply when eGFR is below 60 mL/min/1.73 m^2^. These findings support routine eGFR-guided risk stratification and early intensive monitoring in patients with advanced HF.

## Introduction

Accelerated population aging and improved survival after acute cardiovascular events have driven a steady rise in the prevalence of heart failure (HF) (1). Among patients with HF, renal dysfunction represents a common comorbidity associated with poor prognosis (2). Because HF usually denotes an irreversible structural injury to the myocardium, early identification of individuals at highest risk remains the cornerstone of efforts to forestall cardiovascular death (3). Contemporary epidemiologic data converge on the conclusion that a reduction in estimated glomerular filtration rate (eGFR) independently predicts adverse outcomes in patients with HF, regardless of whether it is associated with acute kidney injury (AKI) or chronic kidney disease (CKD). These adverse outcomes include an increased risk of in-hospital mortality and accelerated progression of cardiorenal dysfunction. Notably, this bidirectional crosstalk is mediated by overlapping pathophysiological pathways, rendering eGFR staging one of the potential key prognostic tools for risk stratification in hospitalized patients with HF (4–6).

The eGFR is a robust, readily available metric of kidney function, widely utilized in clinical practice to evaluate renal filtration capacity. Beyond its primary role in reflecting renal health, eGFR encodes age, sex, nutritional status, and cumulative comorbidity burden, thereby serving as a parsimonious surrogate of global physiological reserve and biological vulnerability (7, 8). A cohort study from a large, integrated healthcare system demonstrated a clear dose–response relationship between CKD stage and cardiovascular risk: each incremental decline in eGFR category translated into a stepwise rise in major cardiovascular events (9). Among our hospitalized HF population, admission eGFR provided prognostic information that extended well beyond renal function *per se*, a utility underscored by its statistically significant association with in-hospital mortality.

A consistent body of evidence links eGFR decline with cardiorenal syndrome (CRS) and adverse short-term outcomes in patients with HF (10). Among adults with CKD, those with an eGFR < 30 mL/min/1.73 m^2^ constitute <0.5% of the general population yet carry the highest burden of morbidity and mortality (11), including initiation of continuous renal-replacement therapy (CRRT), incident cardiovascular disease (CVD), and all-cause mortality (12). A retrospective cohort study from Flanders demonstrated that the leading cause of death in CKD is not end-stage renal disease (ESRD) *per se*, but rather HF and other cardiovascular complications; moreover, a rapid eGFR decline amplifies the subsequent risk of developing HF (13). Compared with individuals with preserved kidney function (eGFR >90 mL/min/1.73 m^2^), those with moderate-to-severe CKD (stage ≥ 3 or eGFR < 60 mL/min/1.73 m^2^) face an approximately 3-fold higher risk of developing HF (14).

Although reduced eGFR has repeatedly been shown to stratify cardiovascular risk, granular data delineating a dose-response relationship between progressively lower eGFR strata and in-hospital mortality among patients with heart failure (HF) remain scarce. This knowledge gap is most pronounced for individuals with advanced CKD (eGFR < 30 mL/min/1.73 m^2^), a population routinely excluded from randomized clinical trials (RCTs) (15, 16). Enhancing the evidence base in this area would facilitate more precise risk stratification and timely identification of high-risk individuals.

## Methods

### Study design and population

This study retrospectively enrolled patients hospitalized for HF at The First Affiliated Hospital of Xinjiang Medical University between January 1, 2009, and December 31, 2024. Patients were stratified into five categories based on their baseline eGFR: ≥90 mL/min/1.73 m^2^, 60–89 mL/min/1.73 m^2^, 45–59 mL/min/1.73 m^2^, 30–44 mL/min/1.73 m^2^, and <30 mL/min/1.73 m^2^. The eGFR was calculated using the Chronic Kidney Disease Epidemiology Collaboration (CKD-EPI) equation, which incorporates patient demographic characteristics and serum creatinine (Scr) levels (17). To ensure data integrity, we excluded patients with incomplete medical records. The study protocol was approved by the Institutional Review Board of the First Affiliated Hospital of Xinjiang Medical University (approval nos. K202403-48 and K202403-48-2503A-Y1). Because all personal identifiers were removed before analysis, the requirement for written informed consent was waived by the same committee. Exclusion criteria were age <18 years, missing inpatient Scr or left ventricular ejection fraction data, maintenance dialysis or prior kidney transplantation, and concurrent malignancy, sepsis, pregnancy, or lactation. The primary end point was all-cause in-hospital mortality.

### Data collection

Baseline characteristics were extracted from the electronic medical record and included age, sex, alcohol consumption, smoking status, medical history (hypertension, diabetes mellitus, coronary heart disease, atrial fibrillation, and active malignancy), systolic blood pressure (SBP) and diastolic blood pressure (DBP), HF-related clinical findings, New York Heart Association (NYHA) class, echocardiographic parameters, and medication history (angiotensin-converting-enzyme inhibitors or angiotensin-receptor blockers, mineralocorticoid receptor antagonists, sodium–glucose cotransporter-2 inhibitors, furosemide, and β-blockers). Fasting venous blood was drawn within 24 h of admission and analyzed on automated platforms (Roche Cobas 8000 and Sysmex XN-2000). Variables recorded were white-cell count (WBC), hemoglobin (Hb), serum creatinine (Scr), uric acid (UA), cystatin (Cys-C), total cholesterol (TC), high- density lipoprotein cholesterol (HDL-C), low-density lipoprotein cholesterol (LDL-C), triglycerides (TG), alanine aminotransferase (ALT), aspartate aminotransferase (AST), total bilirubin (TBIL), albumin (ALB), globulin (GLO), serum sodium (Na⁺), serum potassium (K⁺), and N-terminal pro-brain natriuretic peptide (NT-proBNP).

### Statistical analysis

**Figure 1:**
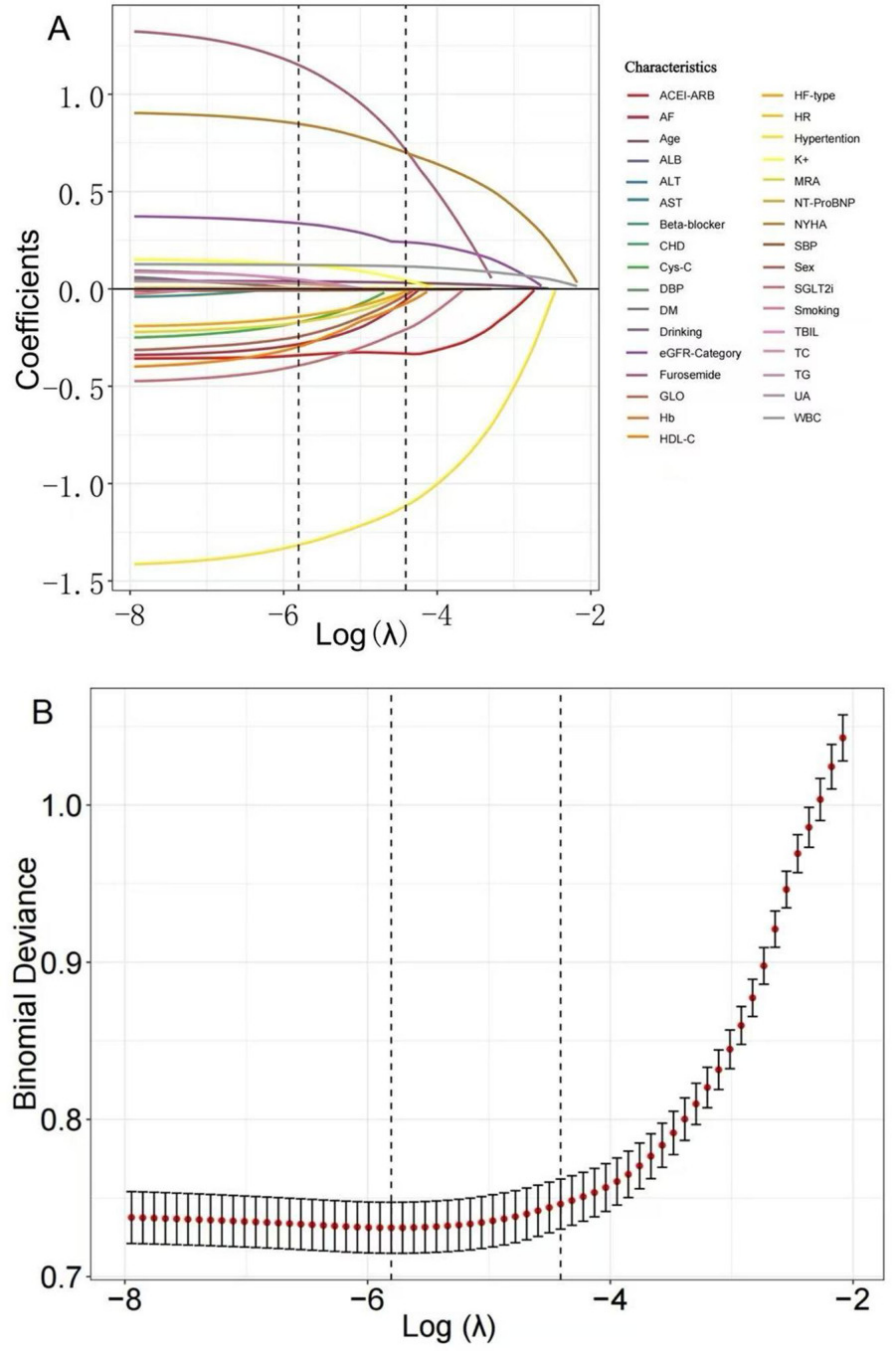
Diagram of coefficient path of each variable in LASSO regression and 10-fold cross-validation plots for LASSO regression. **(A)** Coefficient path of each variable in LASSO regression. **(B)** 10-fold cross-validation plots for LASSO regression.

Data analysis was performed using SPSS version 26.0 (IBM, Armonk, NY) and R 4.3.2. Missing data were addressed via multiple imputation. A two-sided *P* < 0.05 was considered statistically significant. Baseline characteristics of the cohort are presented across the five eGFR strata: continuous variables were presented as mean ± standard deviation if normally distributed, with intergroup comparisons conducted using one-way analysis of variance (ANOVA); for non-normally distributed variables, median (interquartile range, IQR) was used, and intergroup differences were assessed by the Kruskal–Wallis *H* test. Categorical variables were expressed as frequency (percentage), and intergroup comparisons were performed using the Pearson chi-square (*χ*^2^) test. *Post-hoc* pairwise comparisons between groups were adjusted with the Bonferroni correction, and a corrected *P* < 0.05 was considered statistically significant. Baseline variables were first screened with the least absolute shrinkage and selection operator (LASSO) regression; 10-fold cross-validation identified the optimal *λ*, and the *λ*.1se criterion ultimately retain 18 variables. The variable trajectories and selection process of Lasso regression are shown in (Figures 1A,B). Logistic regression models were constructed to quantify the association between eGFR category and in-hospital mortality while adjusting for established confounders. Three sequential models were built: model 1 adjusted for age, sex, heart rate, SBP, DBP; model 2 added hypertension, atrial fibrillation, and use of ACE inhibitors or ARBs, MRAs, and SGLT2 inhibitors; model 3 further adjusted for HF subtype, NYHA class, Hb, HDL-C, and K^+^. Interaction was examined in subgroups defined by sex, age, HF subtype (HFpEF, HFmrEF, HFrEF), NYHA class, and comorbidities; forest plots present adjusted and unadjusted odds ratios with 95% confidence intervals. Restricted cubic splines (RCS) with knots at the 5th, 35th, 65th, and 95th percentiles of baseline eGFR were fitted to evaluate potential non-linear relations with in-hospital mortality.

## Results

### Baseline characteristics

**Figure 2:**
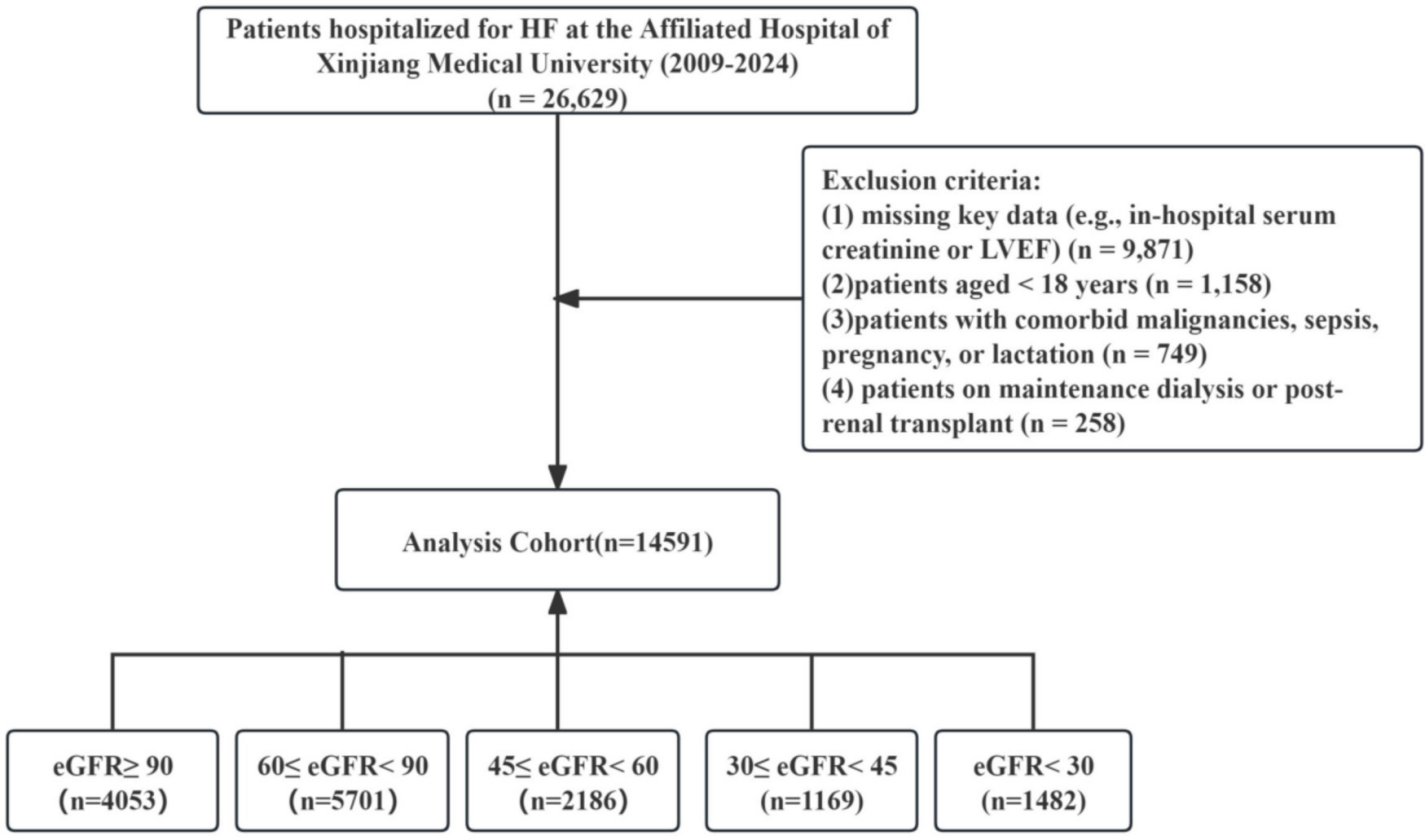
Flowchart of study participants.

A total of 14,591 patients with heart failure were enrolled in the present study. The cohort was stratified into five subgroups according to eGFR levels: eGFR ≥ 90 mL/min/1.73 m^2^ (*n* = 2,053), 60–89 mL/min/1.73 m^2^ (*n* = 5,701), 45–59 mL/min/1.73 m^2^ (*n* = 2,186), 30–44 mL/min/1.73 m^2^ (*n* = 1,169), and <30 mL/min/1.73 m^2^ (*n* = 1,482). Figure 2 depicts the patient enrollment flow chart. The in-hospital mortality rates across the five aforementioned subgroups were 5.77%, 6.37%, 8.19%, 11.72%, and 10.93%, respectively. Table 1 summarizes the baseline profile of each eGFR group. Patients in lower eGFR strata exhibited significantly different profiles across most measured variables compared to those with higher eGFR strata (all *P* < 0.001). Specifically, Those with eGFR < 30 mL/min/1.73 m^2^ had higher SBP, DBP, WBC, Scr, UA, Cys-C, GLO, K⁺, and NT-proBNP, alongside lower Hb, ALB, and HDL-C compared with the higher-eGFR strata. LASSO regression with 10-fold cross-validation identified 18 Characteristics with non-zero coefficients as predictors of in-hospital mortality in patients with HF, including Age, Sex, HR, DBP, SBP, eGFR Category, HF-Type, NYHA class, ACEI-ARB, MRA, SGLT2i, Furosemide, WBC, HDL-C, Hb, K+, Hypertension, AF.

**Table 1:** Demographic and clinical characteristics of study participants.

Characteristics	eGFR Category, mL/min/1.73 m^2^					*P*
	≥90 (*n* = 4,053)	[60–90) (*n* = 5,701)	[45–60) (*n* = 2,186)	[30–45) (*n* = 1,169)	<30 (*n* = 1,482)	
Age, year	57.00 (47.00, 67.00)	64.00 (54.00, 73.00^a^	68.00 (58.00, 76.00)^b^^,^^e^	70.00 (59.00, 78.00)^c^^,^^f^	60.00 (48.00, 73.00)^d^^,^^g^^,^^i^^,^^j^	<0.001
Sex, *n* (%)						
Male	1,780 (43.92)	3,938 (69.08)^a^	1,671 (76.44)^b^^,^^e^	814 (69.63)^c^^,^^h^	992 (66.94)^d^^,^^i^	<0.001
Female	2,273 (56.08)	1,763 (30.92)^a^	515 (23.56)^b^^,^^e^	355 (30.37)^c^^,^^h^	490 (33.06)^d^^,^^i^	
HR, (bpm)	83.00 (76.00, 94.00)	82.00 (75.00, 91.00)	83.00 (75.00, 93.00)^e^	84.00 (75.00, 96.00)^f^	84.00 (78.00, 96.00)^d^^,^^g^^,^^i^^,^^j^	<0.001
SBP, (mmHg)	130.00 (121.00, 140.00)	131.000 (12.00, 141.00)	132.00 (123.00, 141.00)^b^	132.00 (122.00, 141.00)	135.00 (125.00, 147.70)^d^^,^^g^^,^^i^^,^^j^	<0.001
DBP, (mmHg)	75.00 (69.00, 81.00)	75.00 (68.00, 81.00)^a^	75.00 (68.00, 81.00)^b^	74.00 (67.00, 81.00)^c^	77.00 (70.00, 84.00)^d^^,^^g^^,^^i^^,^^j^	<0.001
WBC, (×10^9^/L)	6.97 (5.70, 8.74)	6.97 (5.81, 8.49)	7.14 (5.87, 8.80)^b^^,^^e^	7.46 (6.04, 9.73)^c^^,^^f^^,^^h^	7.53 (5.99, 10.01)^d^^,^^g^^,^^j^	<0.001
Hb, (g/L)	132.00 (118.00, 144.00)	137.00 (124.00, 148.00)^a^	135.00 (120.00, 148.00)^b^^,^^e^	127.00 (111.00, 142.00)^c^^,^^f^^,^^h^	100.00 (82.00, 123.00)^d^^,^^g^^,^^i^^,^^j^	<0.001
Scr, (µmoI/L)	57.00 (50.00, 63.00)	77.97 (71.40, 84.09)^a^	100.45 (94.10, 107.46)^b^^,^^e^	129.14 (119.25, 143.00)^c^^,^^f^^,^^h^	298.56 (207.72, 563.13)^d^^,^^g^^,^^i^^,^^j^	<0.001
eGFR, (mL/min)	100.55 (95.13, 107.46)	74.23 (67.31, 82.23)^a^	53.45 (49.25, 56.79)^b^^,^^e^	39.03 (35.11, 42.22)^c^^,^^f^^,^^h^	14.77 (7.35, 23.02)^d^^,^^g^^,^^i^^,^^j^	<0.001
UA, (μmol/L)	283.00 (225.00, 350.03)	343.25 (282.00, 412.50)^a^	390.95 (326.41, 476.13)^b^^,^^e^	440.00 (362.52, 540.10)^c^^,^^f^^,^^h^	461.41 (373.92, 564.77)^d^^,^^g^^,^^i^	<0.001
Cys-C, (mg/L)	0.96 (0.82, 1.11)	1.10 (0.95, 1.28)^a^	1.32 (1.14, 1.55)^b^^,^^e^	1.68 (1.45, 2.00)^c^^,^^f^^,^^h^	3.13 (2.31, 4.51)^d^^,^^g^^,^^i^	<0.001
ALB, (g/L)	38.20 (33.61, 41.80)	38.40 (34.70, 42.00)^a^	37.50 (33.69, 41.41)^e^	36.08 (31.90, 40.10)^c^^,^^f^^,^^h^	33.14 (28.55, 37.50)^d^^,^^g^^,^^i^^,^^j^	<0.001
GLO, (g/L)	28.90 (25.20, 33.05	28.44 (24.80, 32.20)^a^	28.92 (25.40, 32.74)^e^	29.86 (26.15, 33.80)^c^^,^^f^^,^^h^	30.00 (26.55, 33.89)^d^^,^^g^^,^^i^	<0.001
ALT, (U/L)	22.90 (15.00, 37.40)	23.30 (15.98, 37.40)	22.79 (15.67, 38.10)	22.80 (15.13, 40.67)	21.00 (13.10, 38.80)^d^^,^^g^^,^^i^^,^^j^	<0.001
AST, (U/L)	24.22 (17.73, 36.83)	24.50 (18.30, 35.70)	25.18 (19.20, 37.17)^b^^,^^e^	26.50 (19.31, 44.70)^c^^,^^f^	24.43 (17.24, 41.49)^j^	<0.001
TBIL, (μmol/L)	13.20 (9.33, 18.90)	14.12 (10.25, 20.10)^a^	14.98 (10.71, 21.19)^b^^,^^e^	14.74 (10.10, 21.90)^c^	11.14 (7.53, 17.09)^d^^,^^g^^,^^i^^,^^j^	<0.001
TG, (mmol/L)	1.22 (0.90, 1.78)	1.20 (0.91, 1.68)	1.20 (0.91, 1.71)	1.22 (0.97, 1.75)	1.34 (1.02, 1.97)^d^^,^^g^^,^^i^^,^^j^	<0.001
TC, (mmol/L)	3.75 (3.17, 4.45)	3.72 (3.16, 4.35)	3.64 (3.08, 4.23)^b^^,^^e^	3.69 (3.09, 4.23)^c^	3.68 (3.14, 4.32)	<0.001
HDL-C, (mmol/L)	0.92 (0.76, 1.13)	0.92 (0.77, 1.11)	0.91 (0.76, 1.10)	0.89 (0.74, 1.05)^c^^,^^f^^,^^h^	0.86 (0.70, 1.00)^d^^,^^g^^,^^i^^,^^j^	<0.001
LDL-C, (mmol/L)	2.38 (1.89, 2.91)	2.36 (1.90, 2.86)	2.31 (1.85, 2.76)^b^^,^^e^	2.33 (1.85, 2.72)^c^^,^^f^	2.33 (1.84, 2.81)	<0.001
K^+^, (mmol/L)	3.70 (3.43, 3.99)	3.78 (3.49, 4.05)^a^	3.86 (3.55, 4.17)^b^^,^^e^	3.98 (3.62, 4.34)^c^^,^^f^^,^^h^	4.15 (3.69, 4.71)^d^^,^^g^^,^^i^^,^^j^	<0.001
Na^+^, (mmol/L)	140.00 (137.00, 142.00)	140.00 (138.00, 142.00)^a^	140.00 (137.00, 142.00)^e^	139.00 (136.00, 142.00)^c^^,^^f^^,^^h^	139.00 (136.00, 141.00)^d^^,^^g^^,^^i^	<0.001
NT-ProBNP, (ng/L)	1,377.00 (671.00, 261.00)	1,510.00 (844.00, 3,302.00)^a^	2,110.00 (1,122.00, 4,758.50)^b^^,^^e^	2,720.00 (1,250.00, 5,880.00)^c^^,^^f^^,^^h^	3,481.00 (1,526.75, 7,998.50)^d^^,^^g^^,^^i^^,^^j^	<0.001
Hypertention, *n* (%)	1,401 (34.57)	2,347 (41.17)^a^	1,154 (52.79)^b^^,^^e^	707 (60.48)^c^^,^^f^^,^^h^	1,028 (69.37)^d^^,^^g^^,^^i^^,^^j^	<0.001
DM, *n* (%)	990 (24.43)	1,478 (25.93)	662 (30.28)^b^^,^^e^	434 (37.13)^c^^,^^f^^,^^h^	567 (38.26)^d^^,^^g^^,^^i^	<0.001
CHD, *n* (%)	1,521 (37.53)	2,568 (45.04)^a^	1,063 (48.63)^b^^,^^e^	593 (50.70)^c^^,^^f^	499 (33.67)^g^^,^^i^^,^^j^	<0.001
AF, *n* (%)	657 (16.21)	1,299 (22.79)^a^	551 (25.21)^b^	280 (23.95)^c^	228 (15.38)^g^^,^^i^^,^^j^	<0.001
HF-type, *n* (%)						
HFpEF	2,795 (68.96)	3,422 (60.02)^a^	1,199 (54.85)^b^^,^^e^	654 (55.95)^c^	932 (62.89)^d^^,^^i^	<0.001
HFmrEF	668 (16.48)	1,127 (19.77)^a^	462 (21.13)^b^	276 (23.61)^c^^,^^f^	348 (23.48)^d^^,^^g^	
HFrEF	590 (14.56)	1,152 (20.21)^a^	525 (24.02)^b^^,^^e^	239 (20.44)^c^	202 (13.63)^g^^,^^i^^,^^j^	
NYHA, *n* (%)						
Ⅰ	717 (17.69)	977 (17.14)	417 (19.08)	226 (19.33)	139 (9.38)^d^^,^^g^^,^^i^^,^^j^	<0.001
Ⅱ	1,251 (30.87)	1,684 (29.54)	609 (27.86)	296 (25.32)^c^^,^^f^	352 (23.75)^d^^,^^g^	
Ⅲ	1,606 (39.62)	2,328 (40.83)	835 (38.20)	460 (39.35)	548 (36.98)	
Ⅳ	479 (11.82)	712 (12.49)	325 (14.87)^b^	187 (16.00)^c^^,^^f^	443 (29.89)^d^^,^^g^^,^^i^^,^^j^	
ACEI-ARB, *n* (%)						
YES	2,959 (73.01)	4,617 (80.99)^a^	1,842 (84.26)^b^^,^^e^	956 (81.78)^c^	1,082 (73.01)^g^^,^^i^^,^^j^	<0.001
Beta-blocker, *n* (%)						
YES	3,103 (76.56)	4,581 (80.35)	1,788 (81.79)	887 (75.88)	1,045 (70.51)	<0.001
MRA, *n* (%)						
YES	2,696 (66.52)	4,120 (72.27)^a^	1,595 (72.96)^b^	814 (69.63)	609 (41.09)^d^^,^^g^^,^^i^^,^^j^	<0.001
SGLT2i, *n* (%)						
YES	759 (18.73)	1,241 (21.77)^a^	477 (21.82)^b^	213 (18.22)	157 (10.59)^d^^,^^g^^,^^i^^,^^j^	<0.001
Furosemide, *n* (%)						
YES	3,192 (78.76)	4,670 (81.92)^a^	1,881 (86.05)^b^^,^^e^	1,057 (90.42)^c^^,^^f^^,^^h^	1,274 (85.96)^d^^,^^g^^,^^j^	<0.001
Smoking, *n* (%)						
YES	1,132 (27.93)	2,022 (35.47)^a^	760 (34.77)^b^	357 (30.54)^f^	460 (31.04)^g^	<0.001
Drinking, *n* (%)						
YES	667 (16.46)	1,243 (21.80)^a^	469 (21.45)^b^	234 (20.02)^c^	284 (19.16)	<0.001
Death, *n* (%)						
YES	234 (5.77)	363 (6.37)	179 (8.19)^b^^,^^e^	137 (11.72)^c^^,^^f^^,^^h^	162 (10.93)^d^^,^^g^	<0.001

HR, heart rate; SBP, systolic blood pressure; DBP, diastolic blood pressure; WBC, white blood cell; Hb, hemoglobin; Scr, serum creatinine; eGRF, estimated glomerular filtration rate; UA, uric acid; CysC, cystatinC; ALB, albumin; GLO, globulin; ALT, alanine transaminase; AST, aspartate aminotransferase; TBIL, total bilirubin; TG, triglyceride; TC, total cholesterol; HDL-C, high-density lipoprotein cholesterol; LDL-C, low-density lipoprotein cholesterol; K^+^, serum potassium; Na^+^, serum sodium; NT-ProBNP, N-terminal pro-brain natriuretic peptide; DM, diabetes; CHD, coronary heart disease; AF, atrial fibrillation; HF-Type, heart failure type; HFmrEF, heart failure with mid-range ejection fraction; HFpEF, heart failure with preserved ejection fraction; HFrEF, heart failure with reduced ejection fraction; NYHA, New York Heart Association; ACEI/ARB, angiotensin converting enzyme inhiiitors/angiotensin receptor blocker; MRA, mineralocorticoid receptor antagonist; SGLT2i, sodium-dependent glucose transporters 2 inhibitors.

a Statistically significant differences (*P* < 0.05) were found between eGFR ≥ 90 and 60 ≤ eGFR < 90.

b Statistically significant differences (*P* < 0.05) were found between eGFR ≥ 90 and 45 ≤ eGFR < 60.

c Statistically significant differences (*P* < 0.05) were found between eGFR ≥ 90 and 30 ≤ eGFR < 45.

d Tatistically significant differences (*P* < 0.05) were found between eGFR ≥ 90 and eGFR < 30.

e Statistically significant differences (*P* < 0.05) were found between 60 ≤ eGFR < 90 and 45 ≤ eGFR < 60.

f Statistically significant differences (*P* < 0.05) were found between 60 ≤ eGFR < 90 and 30 ≤ eGFR < 45.

g Statistically significant differences (*P* < 0.05) were found between 60 ≤ eGFR < 90 and eGFR < 30.

h Statistically significant differences (*P* < 0.05) were found between 45 ≤ eGFR < 60 and 30 eGFR < 45.

i Statistically significant differences (*P* < 0.05) were found between 45 ≤ eGFR < 60 and eGFR < 30.

j Statistically significant differences (*P* < 0.05) were found between 30 ≤ eGFR < 45 and eGFR < 30.

### Association of eGFR with in-hospital mortality

**Figure 3:**
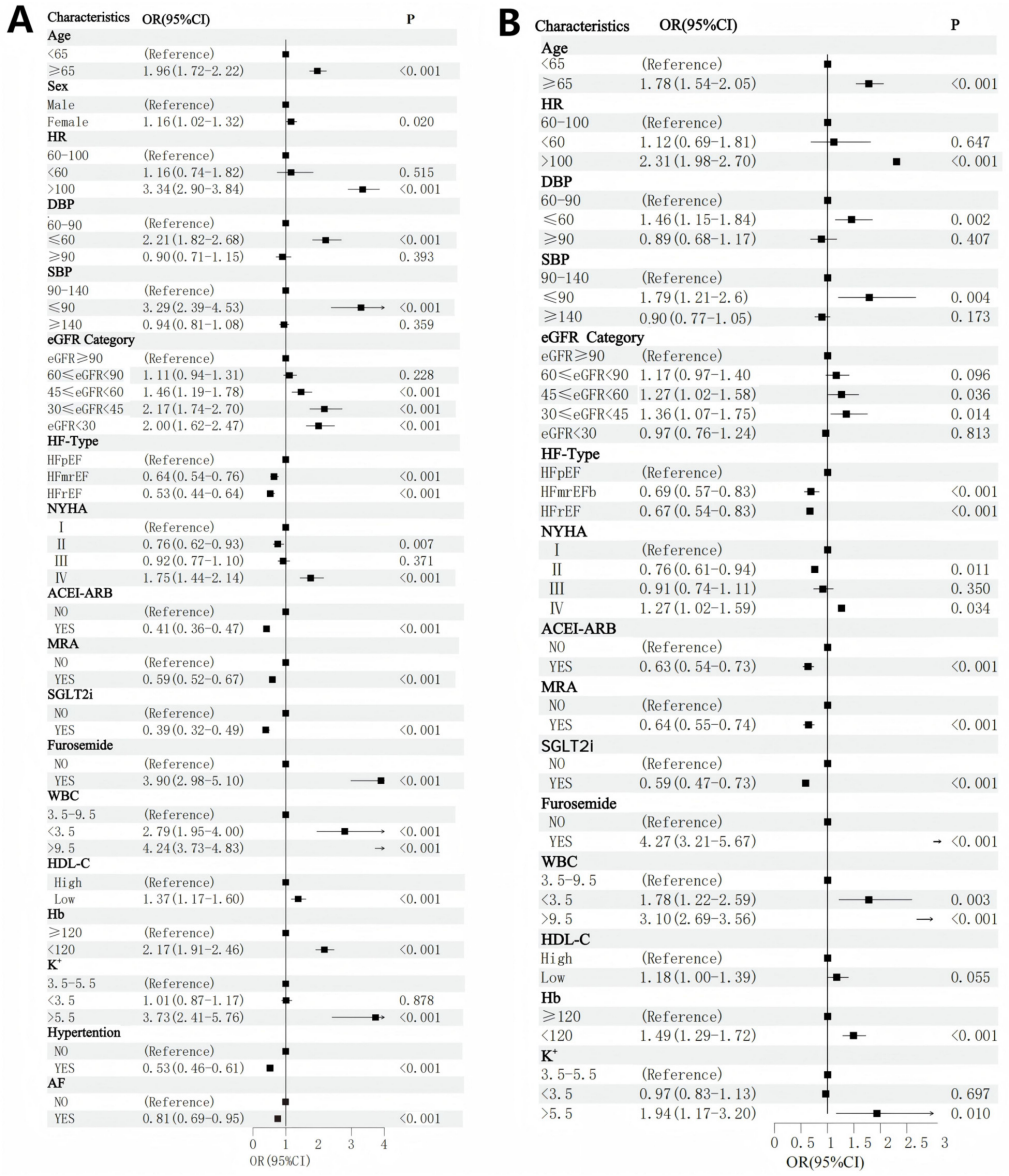
Results of logistic regression for patients. **(A)** Univariate logistic regression. **(B)** Multivariate logistic regression.

The 18 variables identified via least LASSO regression were individually subjected to univariate analysis as independent variables Figure 3A. Subsequently, the variables filtered by univariate analysis were incorporated into a multivariate logistic regression model, with non-statistically significant variables excluded using the bidirectional stepwise elimination method Table 2 is the variable assignment table. Results of the multivariate logistic regression analysis showed that Figure 3B, compared with the eGFR ≥ 90 mL/min/1.73 m^2^ group, the eGFR 45–59 mL/min/1.73 m^2^ group had a 27% increased risk of in-hospital mortality (OR 1.27, 95% CI 1.02–1.58, *P* = 0.036), and the risk of in-hospital mortality was further elevated to 36% in the eGFR 30–44 mL/min/1.73 m^2^ group (OR 1.36, 95% CI 1.07–1.75, *P* = 0.014). These findings indicate that moderate to severe CKD (eGFR < 60 mL/min/1.73 m^2^) is one of the independent risk factors for in-hospital mortality in patients hospitalized with HF, and the risk increases with the deterioration of renal function. Beyond renal function, age ≥65 years, HR ≥100 beats per minute, hypotension, low hemoglobin, and hyperkalemia were also independent risk factors for in-hospital mortality in HF patients.

**Table 2:** Variable assignment table.

Variables	Variable assignment
Age	<65 = 0, ≥65 = 1
Sex	Male = 0, Female = 1
HR	[60, 100] = 0, <60 = 1, >100 = 2
SBP	(90, 140) = 0, ≤90 = 1, ≥140 = 2
DBP	(60, 90) = 0, ≤60 = 1, ≥90 = 2
WBC	[3.5, 9.5] = 0, <3.5 = 1, >9.5 = 2
Hb	≥120 = 0, <120 = 1
HDL-C	High (Male ≥ 1.0, Female ≥ 1.3) = 0, Low (Male < 1.0, Female < 1.3) = 1
K^+^	[3.5, 5.5] = 0, <3.5 = 1, >5.5 = 2
eGFR Category	≥90 = 0, [60–90) = 1, [45–60) = 2, [30–45) = 3, <30 = 4
HF-Type	HFpEF (LVEF ≥ 50%) = 0, HFmrEF (LVEF 40%–49%) = 1, HFrEF (LVEF < 40%) = 2
NYHA	I = 1, II = 2, III = 3, IV = 4
Hypertension	NO = 0, YES=1
AF	NO = 0, YES=1
ACEI/ARB	NO = 0, YES=1
MRA	NO = 0, YES=1
SGLT2i	NO = 0, YES=1
Furosemide	NO = 0, YES=1
In-hospital mortality	NO = 0, YES=1

Three stepwise adjusted logistic models are summarised in Table 3. Across all models, lower eGFR was associated with a graded increase in the risk of in-hospital death. In the unadjusted model, patients with eGFR 45–59, 30–44 and <30 mL/min/1.73 m^2^ had significantly higher mortality than those with eGFR ≥90 mL/min/1.73 m^2^ (OR 1.46, 95% CI 1.19–1.78, *P* < 0.001; OR 2.17, 95% CI 1.74–2.70, *P* < 0.001 and OR 2.00, 95% CI 1.62–2.47, *P* < 0.001, respectively). After adjustment for age, sex, HR, SBP, DBP (Model 1), eGFR 30–44 and <30 mL/min/1.73 m^2^ remained independently associated with mortality (OR 1.63, 95% CI 1.29–2.06, *P* < 0.001 and OR 1.76, 95% CI 1.41–2.19, *P* < 0.001). Further adjustment for medication history and comorbidities (Model 2) revealed that the increased mortality risks persisted in patients with eGFR 45–59, 30–44, and <30 mL/min/1.73 m^2^, with (OR 1.28, 95% CI 1.04–1.59, *P* = 0.023; OR 1.56, 95% CI 1.23–1.98, *P* < 0.001 and OR 1.35 95% CI 1.08–1.70, *P* = 0.008, respectively). In the final fully adjusted model (Model 3), additional variables including HF subtype, NYHA class, Hb, HDL-C, and K⁺ were incorporated. The risk remained elevated for eGFR 45–59 and 30–44 mL/min/1.73 m^2^ (OR 1.27, 95% CI 1.02–1.58; *P* = 0.036 and OR 1.36, 95% CI 1.07–1.75; *P* = 0.014). Decreased eGFR was associated with an increased risk of in-hospital mortality in patients with HF, and this association was more pronounced when eGFR was below 45 mL/min/1.73 m^2^. However, the association in the eGFR < 30 mL/min/1.73 m^2^ subgroup was attenuated and no longer statistically significant after full adjustment in Model 3 (OR 0.97, 95% CI 0.76–1.24, *P* = 0.813). These findings indicate that eGFR may serve as an independent prognostic factor for patients with HF, particularly in those with eGFR < 60 mL/min/1.73 m^2^, suggesting that eGFR levels should be integrated into clinical assessment.

**Table 3:** Multivariable adjusted odds ratio (95% confidence intervals) of eGFR category for in-hospital morality.

eGFR Category	Crude		Model 1		Model 2		Model 3	
	OR (95% CI)	*P*	OR (95% CI)	*P*	OR (95% CI)	*P*	OR (95% CI)	*P*
eGFR ≥ 90	(Reference)		(Reference)		(Reference)		(Reference)	
60 ≤ eGFR < 90	1.11 (0.94–1.31)	0.228	1.01 (0.85–1.21)	0.870	1.09 (0.92 ∼1.31)	0.317	1.17 (0.97–1.40)	0.096
45 ≤ eGFR < 60	1.46 (1.19–1.78)	<0.001	1.20 (0.97–1.49)	0.099	1.28 (1.04 ∼1.59)	0.023	1.27 (1.02–1.58)	0.036
30 ≤ eGFR < 45	2.17 (1.74–2.70)	<0.001	1.63 (1.29–2.06)	<0.001	1.56 (1.23–1.98)	<0.001	1.36 (1.07–1.75)	0.014
eGFR < 30	2.00 (1.62–2.47)	<0.001	1.76 (1.41–2.19)	<0.001	1.35 (1.08∼ 1.70)	0.008	0.97 (0.76–1.24)	0.813

OR, odds ratio; CI, confidence interval; Model 1, adjusted for age, gender, HR, SBP, DBP; Model 2, adjusted for Model 1 + ACEI-ARB, MRA, SGLT2i, furosemide, hypertention, AF; Model 3, adjusted for Model 2 + HF-Type, NYHA, WBC, Hb, HDL-C, K+.

### Subgroup analysis of eGFR and in-hospital mortality

**Figure 4:**
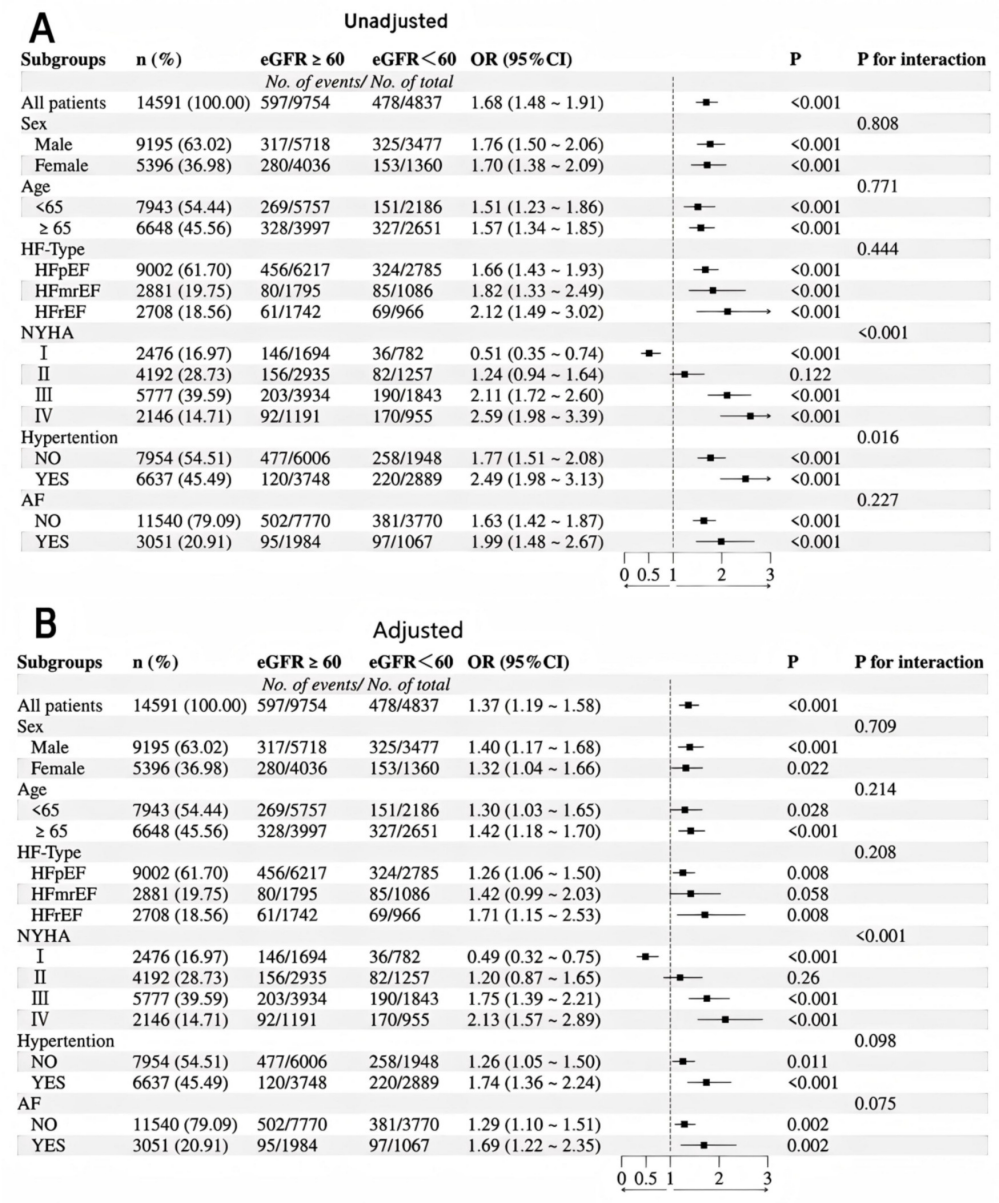
Subgroub analyses of the different eGFR groupes. **(A)** Unadjusted odds ratios (OR) for subgroup analyses. **(B)** Adjusted aOR (all covariates in the final multivariable logistic regression model, Model 3) for subgroup analyses.

Subgroup analysis revealed that in the unadjusted model, eGFR < 60 mL/min/1.73 m^2^ was significantly associated with an increased risk of mortality in the overall population (OR 1.68, 95% CI 1.48–1.91, *P* < 0.001). A consistent trend of elevated risk was observed across all subgroups, which was particularly pronounced in patients with HFrEF (OR = 2.12, *P* < 0.001), NYHA class IV (OR = 2.59, *P* < 0.001), and those with comorbid hypertension (OR = 2.49, *P* < 0.001). Interaction analysis indicated that both NYHA class and hypertension significantly modified the association between eGFR and outcome (*P* for interaction <0.05 for each). After full adjustment for all covariates identified by the final multivariable logistic regression model (Model 3), eGFR < 60 mL/min/1.73 m^2^ remained independently predictive of death (aOR 1.37, 95% CI 1.19–1.58, *P* < 0.001). The interaction with NYHA class remained highly significant (*P* for interaction <0.001), with the prognostic impact of eGFR < 60 mL/min/1.73 m^2^ amplifying stepwise across NYHA class (NYHA III: aOR 1.75; NYHA IV: aOR 2.13). Elevated mortality risk persisted in most subgroups, including male, aged ≥ 65 years, and HFrEF patients (Figures 4A,B).

### Dose-response relationship between eGFR and in-hospital mortality

**Figure 5:**
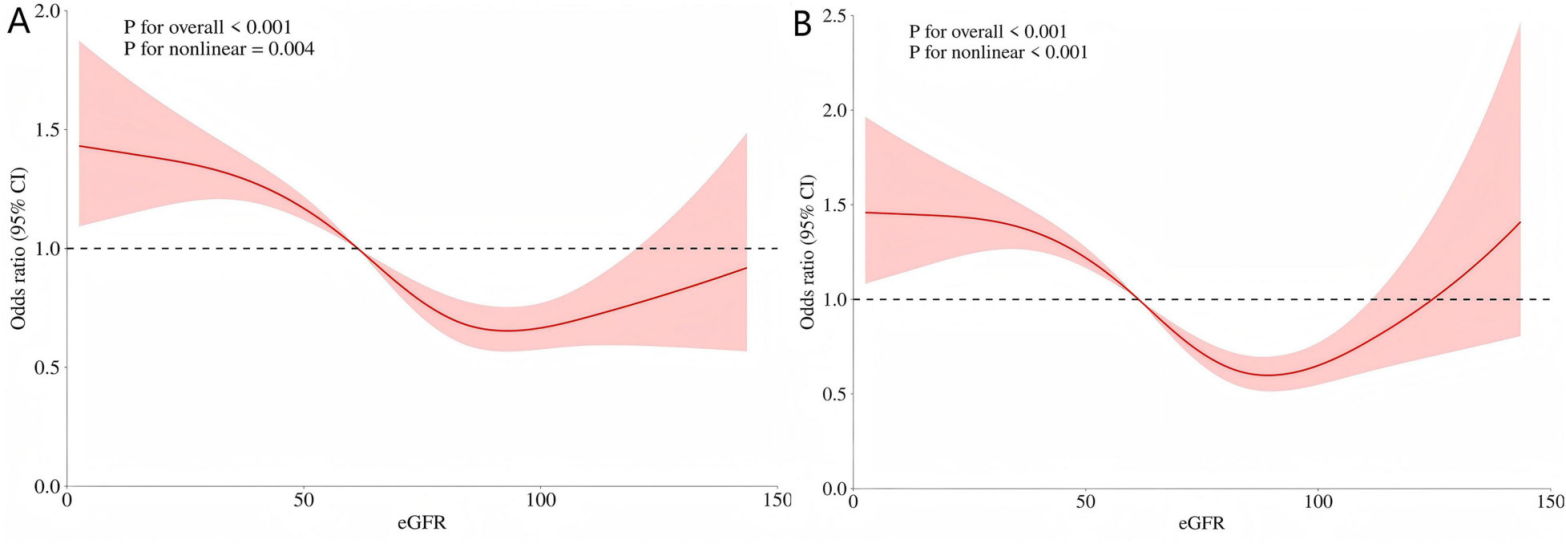
RCS curve of eGFR and inhospital mortality in all patients. **(A)** Unadjusted RCS curve. **(B)** RCS curve adjusted for all covariates in the final multivariable logistic regression model (Model 3).

To further investigate the potential nonlinear association between eGFR and in-hospital mortality risk in patients with HF, an RCS model was employed. The results revealed an optimal threshold of approximately 60 mL/min/1.73 m^2^. The unadjusted RCS analysis Figure 5A showed a significant nonlinear relationship between eGFR levels and in-hospital mortality risk (overall *P* < 0.001, nonlinear *P* = 0.004). When eGFR fell below approximately 60 mL/min/1.73 m^2^, mortality risk exhibited a pronounced upward trend with declining eGFR, indicating that worsening renal function within this range significantly and adversely affected mortality risk. After full adjustment for all covariates, the RCS curve (Figure 5B) demonstrated that the nonlinear association between eGFR and in-hospital mortality risk remained significant (overall *P* < 0.001, nonlinear *P* < 0.001). These findings further confirm that eGFR is an independent risk factor for in-hospital mortality in patients with heart failure, and its impact is not linear across the entire range of renal function.

## Discussion

In this retrospective cohort study, lower eGFR was independently and inversely associated with in-hospital mortality among patients with heart failure, whereas higher eGFR levels correlated with a reduced risk. After full adjustment for potential confounders in Model 3, the mortality risk was 1.36 times higher in the lower eGFR group compared with the reference group. Furthermore, the study observed significant interaction effects between eGFR and NYHA class, as well as a history of hypertension, on the risk of in-hospital mortality. Specifically, patients with lower eGFR exhibited a more pronounced mortality risk in the subgroups of NYHA class Ⅲ–Ⅳ or with hypertension. This association remained statistically significant in patients with NYHA class Ⅲ–Ⅳ after adjustment for confounders. In addition, the RCS model revealed a nonlinear association between eGFR and in-hospital mortality in patients with HF. When eGFR levels were below approximately 60 mL/min/1.73 m^2^, the mortality risk showed a marked upward trend as eGFR declined. This nonlinear association remained significant after further adjustment for potential confounders.

This study further confirms that reduced eGFR is significantly associated with an increased risk of in-hospital mortality in patients with HF, consistent with previous reports demonstrating a close relationship between renal impairment and adverse prognosis (10, 18). We observed that in-hospital mortality was numerically similar between the two renal dysfunction subgroups (eGFR 30–44 mL/min/1.73 m^2^ and eGFR < 30 mL/min/1.73 m^2^). However, in the fully adjusted model for confounders, the association between eGFR < 30 mL/min/1.73 m^2^ and in-hospital mortality did not reach statistical significance. Although numerous prior studies have established that severe renal dysfunction predicts long-term adverse outcomes in HF, evaluating the benefit of risk factor intervention in this population remains challenging (4–6). Löffler et al. (19) reported that although HF patients with the most impaired baseline renal function exhibited worse prognosis, worsening renal function was not independently associated with follow-up endpoints after multivariable adjustment (*P* > 0.05). A plausible explanation is that this subgroup comprised patients with the most severe renal dysfunction, who carried a higher risk of acute kidney injury, a greater comorbid burden, more critical clinical status at admission, and more frequent use of loop diuretics (3, 20). In contrast, patients with relatively higher eGFR were more likely to receive guideline-directed medical therapies, including ACEi/ARB, SGLT2i, or MRA (21, 22). These findings indicate that the severity of renal dysfunction not only directly impacts the therapeutic response in HF but also acts as a key determinant of mortality risk (23, 24). Thus, among patients with extremely severe renal dysfunction (eGFR < 30 mL/min/1.73 m^2^), eGFR itself may lose its ability to independently predict mortality risk. Their prognosis is more strongly determined by the dynamic changes in volume status and the overall condition of multiple organ failure, and the association between this indicator and the mortality risk of HF patients requires cautious interpretation (25). However, In contrast to prior studies characterized by limited sample sizes, relatively outdated therapeutic regimens, and the frequent use of the less accurate Modification of Diet in Renal Disease (MDRD) equation for eGFR estimation (14), the present study leverages the CKD-EPI formula for eGFR estimation and stratifies analyses by age, NYHA class, and HF subtype, thereby providing a more granular and reliable appraisal of renal disease burden and its clinical consequences in hospitalized patients with HF.

Reduced eGFR was significantly associated with increased risks of multiple adverse outcomes (4, 9, 10, 18), with this adverse prognostic effect particularly pronounced among patients with HFrEF, NYHA class III–IV, and concomitant hypertension. However, multivariable logistic regression analysis revealed that HFrEF/HFmrEF was associated with lower in-hospital mortality risk. This seemingly paradoxical finding does not suggest that reduced LVEF *per se* is protective, but rather is more likely attributable to confounding factors, including disease severity, comorbidity burden, differences in the use of guideline-directed medical therapy, and the complexity of HF management in real-world practice (26). In clinical practice, patients with HFrEF/HFmrEF often receive more standardized and intensive guideline-directed medical therapy, thereby achieving substantial survival benefits (27, 28). In addition, although previous studies have reported a higher prevalence of CKD in patients with HFpEF, its impact on prognosis appears relatively weaker in this population (29). Patients with HFpEF are generally older and carry a heavier burden of comorbidities, including non-cardiac conditions such as hypertension, AF, CKD, and frailty, such that their mortality risk may be driven to a greater extent by systemic factors (28, 30). These factors may not be fully captured or adjusted for in routine clinical data, thereby highlighting the relative advantage of the HFrEF/HFmrEF group in the present analysis. Accordingly, these findings do not refute the distinct underlying pathophysiological mechanisms of different heart failure subtypes, but rather indicate that systematic differences exist in treatment intensity and baseline risk across subtypes in contemporary clinical practice, which may influence the comparison of their in-hospital outcomes.

This study further demonstrated that, after multivariable adjustment for age, NYHA class, HR, HF subtype, and eGFR, decreases in both SBP and DBP remained independently associated with adverse clinical outcomes. Higher baseline blood pressure was associated with lower risk of adverse events, a finding consistent with the “blood pressure paradox”—wherein hypertension, traditionally regarded as a cardiovascular risk factor, appears to confer a survival advantage in the setting of HF (31–34). Gheorghiade et al. (31) found that low blood pressure at admission independently predicted mortality among patients with HF, underscoring the intimate link between blood pressure and both short- and long-term survival. Similarly, Böh et al. (32) demonstrated that HF patients with lower SBP had worse clinical outcomes and a significantly higher rate of the primary composite endpoint. Previous studies have also indicated that in lower initial blood pressure in HFrEF patients correlated with an increased incidence of all-cause death and composite clinical events, including HF readmission or all-cause death. SBP < 120 mmHg and DBP < 80 mmHg are independent risk factors for all-cause mortality, increasing the risk by 1.81-fold and 2.24-fold, respectively (34). These findings further support the aforementioned “blood pressure paradox,” whereby conventional hypertension is associated with a survival benefit in HFrEF, which is closely related to the unique pathophysiology of HF. From a pathophysiological standpoint, lower DBP may lead to diminished coronary perfusion, thereby exacerbating myocardial injury and accelerating the progression of left ventricular dysfunction (35). In contrast, an SBP ≤ 90 mmHg could impair renal hemodynamics, promoting the deterioration of renal function and consequently contributing to the cumulative risk of adverse clinical events. These findings collectively suggest that in clinical practice, HF patients with low DBP and SBP warrant close monitoring and early targeted intervention.

Although NT-proBNP is a classic biomarker for prognostic assessment in HF, it was excluded by LASSO regression under the optimal penalty parameter (lambda.min) and subsequent multivariate regression analysis in this study. This finding appears to be inconsistent with previous studies that highlighted NT-proBNP as an independent predictor of short- and long-term mortality in patients with HF (36, 37). However, we posit that this discrepancy does not diminish the established clinical utility of NT-proBNP, but rather suggests that for short-term, highly selective in-hospital outcomes, and in the context of substantial collinearity between NT-proBNP and variables such as eGFR category, NYHA functional class, and age, the variables ultimately included in the study may have greater predictive efficacy for this short-term outcome.

The heart and kidneys exhibit tight pathophysiological crosstalk, which is mediated primarily through the following mechanisms: hemodynamic perturbations leading to renal venous congestion and volume overload (38), activation and dysregulation of the neuroendocrine system, malnutrition and cachexia-related metabolic derangements (39), as well as shared cardiovascular risk factors common to both organs. Among these, hemodynamic change is the pivotal node. Impaired cardiac function raises central venous pressure, producing renal venous stasis, a fall in renal blood flow and a consequent drop in eGFR, which in turn amplifies salt and water retention and perpetuates a vicious cycle. Neuro-endocrine pathways are equally important: concurrent cardiac and renal dysfunction trigger over-activation of the renin–angiotensin–aldosterone system (RAAS) and the sympathetic nervous system, promoting vasoconstriction, fluid retention and direct fibrogenic signalling within both myocardium and renal parenchyma. Chronic systemic inflammation and anemia act as additional common denominators cementing the cardio-renal connection (40, 41).

The study findings provide important insights for the clinical management of HF patients. Monitoring renal function and implementing strategies to preserve and improve eGFR should be a core component of comprehensive patient care. Given the significant association between eGFR levels and mortality, interventions aimed at maintaining renal function should be prioritized in clinical practice. These include optimizing volume management, avoiding nephrotoxic agents, and actively treating comorbidities that contribute to renal function deterioration (42). Renal dysfunction in patients with HF is highly heterogeneous with complex pathophysiological mechanisms, and reliance on eGFR alone cannot fully reflect renal function status or prognostic risk. Substantial evidence indicates that albuminuria (43), BUN or BUN/creatinine ratio, and tubular injury biomarkers (44) provide incremental prognostic information beyond eGFR, enabling more refined assessment of mortality risk in patients with HF. For instance, Testani et al. (45) demonstrated that an elevated BUN/creatinine ratio was associated with worse outcomes, independent of eGFR. Thus, although our findings corroborate eGFR as a critical tool for risk stratification, future research integrating these multidimensional renal assessment frameworks may further optimize risk stratification strategies for hospitalized patients with HF. Furthermore, the significant impact of demographic and clinical factors on prognosis underscores the necessity for implementing individualized treatment strategies. For instance, patients who are older, have a poorer NYHA functional class, and harbor more comorbidities may require closer follow-up and more targeted therapeutic regimens to improve their clinical outcomes.

## Limitations

While the cohort size and granular staging of renal dysfunction strengthen internal validity, several caveats merit emphasis. First, as a descriptive real-world study, it was not designed to definitively establish causal direction and mechanisms. Second, the lack of urinary protein or other kidney-specific biomarkers for more accurate assessment of eGFR may have constrained our findings related to eGFR. While knowledge of these variables would not alter the direction of the relationship between eGFR and the absolute risk of HF outcomes, it could influence the magnitude of this association. despite our efforts to adjust for known confounders, the influence of unmeasured or unrecorded confounding variables cannot be ruled out. Finally, given that this is a single-center study, the generalizability of our findings to broader populations may be limited. In the future, it will be necessary to conduct multicenter studies to validate these results, ensure their applicability across diverse patient populations, and explore potential interventions aimed at improving renal function and reducing mortality in this high-risk group.

## Conclusion

In hospitalized HF patients, eGFR independently predicted increased in-hospital mortality in a dose-dependent manner, with mortality risk rising steeply at eGFR levels below 60 mL/min/1.73 m^2^. This prognostic association was amplified in patients with NYHA class Ⅲ/Ⅳ. Admission eGFR is a critical prognostic marker in this population, mandating intensified clinical management for patients with eGFR < 60 mL/min/1.73 m^2^, especially those with severe HF symptoms.

## Data Availability

The raw data supporting the conclusions of this article will be made available by the authors, without undue reservation.
